# The Duration of Postoperative Antibiotics in Autologous Breast Reconstruction: A Systematic Review and Meta-Analysis

**DOI:** 10.7759/cureus.40631

**Published:** 2023-06-19

**Authors:** Ameer Aldarragi, Nima Farah, Christian M Warner, Ali M Ghasemi, Oghenetega T Ekakitie, Yamen Jabr, Shafiq Rahman

**Affiliations:** 1 Trauma and Orthopaedics, Manchester University NHS Foundation Trust, Manchester, GBR; 2 Plastic Surgery, Leeds General Infirmary, Leeds, GBR; 3 Trauma and Orthopaedics, Stepping Hill Hospital, Stockport NHS Foundation Trust, Stockport, GBR; 4 Trauma and Orthopaedics, Manchester Shoulder and Elbow Unit, Wythenshawe Hospital, Manchester University NHS Foundation Trust, Manchester, GBR; 5 Otolaryngology, Doncaster Royal Infirmary, Leeds, GBR; 6 Plastic Surgery, Pinderfields Hospital, Wakefield, GBR; 7 Trauma and Orthopaedics, Countess of Chester Hospital, Chester, GBR; 8 Plastic Surgery, Sheffield Teaching Hospitals NHS Foundation Trust, Northern General Hospital, Sheffield, GBR

**Keywords:** perioperative antibiotic prophylaxis, surgical site infection (ssi), autologous breast reconstruction, reconstructive breast surgery, oncoplastic breast surgery

## Abstract

Although prophylactic antibiotic use following autologous breast reconstruction post-mastectomy is a common practice, there is no consensus in the literature regarding its duration. Antibiotic stewardship is important to minimise multi-resistant organisms as well as mitigate the associated side effects. Currently, there are no published guidelines regarding the duration of prophylactic antibiotics in autologous breast reconstruction surgery following mastectomy. The authors searched the online literature regarding the administration of antibiotics for autologous breast reconstruction surgery post-mastectomy. The Preferred Reporting Items for Systematic Reviews and Meta-Analysis guidelines were followed. The primary outcome measure was the incidence of surgical site infections (SSIs). Three studies met the inclusion criteria and included a total of 1,400 patients. Overall, 101 (7.2%) SSIs were observed. There was no significant difference in the rate of SSIs when comparing the use of antibiotics for less than or longer than 24 hours postoperatively (odds ratio = 1.434, p = 0.124). There is no significant difference between SSIs with the use of antibiotics for longer than 24 hours when compared to less than 24 hours. Further studies in the form of randomised controlled trials are required to assess the effects of prophylactic antibiotic duration in autologous breast reconstruction following mastectomy.

## Introduction and background

Breast reconstruction post-mastectomy has reported improved psychological outcomes [1], with autologous abdominal tissue becoming increasingly common in specialist UK practice [2]. It is more robust, natural in appearance, and carries lower morbidity with the administration of adjuvant radiotherapy [3]. Breast reconstruction, considered to be a clean surgery, still reports a higher rate of surgical site infections (SSIs) compared to similar cases [4,5], which can lead to poor patient outcomes. The paucity of guidelines on the duration of postoperative antibiotic treatment in breast reconstruction has led to a large discrepancy in clinical practice [6]. In accordance with the Centers for Disease Control and Prevention and the Surgical Care Improvement Project, a maximum of 24 hours of perioperative antibiotics are recommended for clean surgeries [7]. However, reports of longer durations have been recorded within the literature, and the variation in practice among surgeons [8] has generated multiple reviews to provide more defined guidance. A recent meta-analysis by Hai et al. [9] has suggested that extended prophylactic antibiotics do not significantly reduce the incidence of SSIs in breast reconstruction post-mastectomy; however, the majority of the included studies consisted of implant-based reconstructions. Similarly, Phillips et al. [6] have recorded comparable results, but the primary focus of the review was also implant-based reconstruction. Prolonged antibiotic prophylaxis without clinical evidence carries inherent drawbacks such as increased hospital costs, the development of multi-resistant organisms, as well as *Clostridium difficile* infections [10]. Currently, the literature is devoid of a consensus on prophylactic antibiotic duration in autologous breast reconstruction post-mastectomy. The authors aim to report the first systematic review and meta-analysis on this topic to enhance the current evidence base and make recommendations for further research.

## Review

Methods

A systematic review and meta-analysis was performed according to the Preferred Reporting Items for Systematic Reviews and Meta-Analyses (PRISMA) guidelines.

Eligibility Criteria

All cohort, observational, and randomised control studies comparing the effects of antibiotic duration on SSIs in autologous breast reconstruction post-mastectomy were included. The intervention of interest was antibiotic administration for more than 24 hours, and the comparator group included antibiotic prophylaxis less than or equal to 24 hours. Only articles with the aforementioned groups for comparison were included. Case reports, case series, abstracts, and commentaries were not included. Articles not published in the English language were also excluded.

Primary Outcome

The primary outcome was the presence of SSIs. The authors assessed the pattern and usage of prophylactic antibiotics across the selected studies, and the presence of SSIs post-mastectomy autologous breast reconstruction was analysed.

Literature Search Strategy

Two authors SR and YJ independently searched the following electronic databases: Medline, Scopus Preview, Google Scholar, and the Cochrane Central Register of Controlled Trials (CENTRAL). The last search was run on 27 May 2023. There was no restriction on the year of publication in the author’s selection process with all articles considered from any time. The search terminologies included ‘autologous breast reconstruction’, ‘antibiotics’, ‘post mastectomy’, ‘SSI’, ‘surgical site infection’, and ‘infection’. The bibliographic lists of relevant articles were also reviewed.

Selection of Studies

The title and abstract of articles identified from the literature searches were assessed independently by two authors SR and YJ. The full texts of relevant reports were retrieved and the articles that met the eligibility criteria of our review were selected. Any discrepancies in study selection were resolved by discussion between the authors.

Data Extraction and Management

In line with Cochrane’s data collection form for intervention reviews, an electronic data extraction spreadsheet was created and pilot tested in randomly selected articles and adjusted accordingly. Our data extraction spreadsheet included study-related data (first author, year of publication, country of origin of the corresponding author, journal in which the study was published, study design, study size, clinical condition of the study participants, type of breast reconstruction, usage, and duration of antibiotic), baseline demographics of the included populations (age and gender), and primary outcome data (SSIs). Two authors CW and AA cooperatively collected and recorded the results, and any disagreements were resolved via discussion.

Data Synthesis

Open Metanalyst software was used for data synthesis. The extracted data from at least three studies were entered into Open Metanalyst by two independent authors AA and CA. The analysis involved was based on the random-effects model. The results were reported as forest plots with 95% confidence intervals (CIs).

For dichotomous outcome variables, the odds ratio (OR) was used as the summary measure. The OR is the odds of an event or SSI in each group of patients, namely, those who received antibiotics for ≤24 hours and those who received antibiotics for >24 hours. An OR of less than 1 for the infection rate would favour the >24-hour group, and an OR of more than 1 would favour the ≤24-hour group.

Assessment of Heterogeneity

Heterogeneity among the studies was assessed using the Cochran Q test (chi-square). Inconsistency was quantified by calculating I^2^ and interpreted using the following guide: 0% to 25% may represent low heterogeneity, 25% to 75% may represent moderate heterogeneity, and 75% to 100% may represent high heterogeneity.

Results

**Figure 1:**
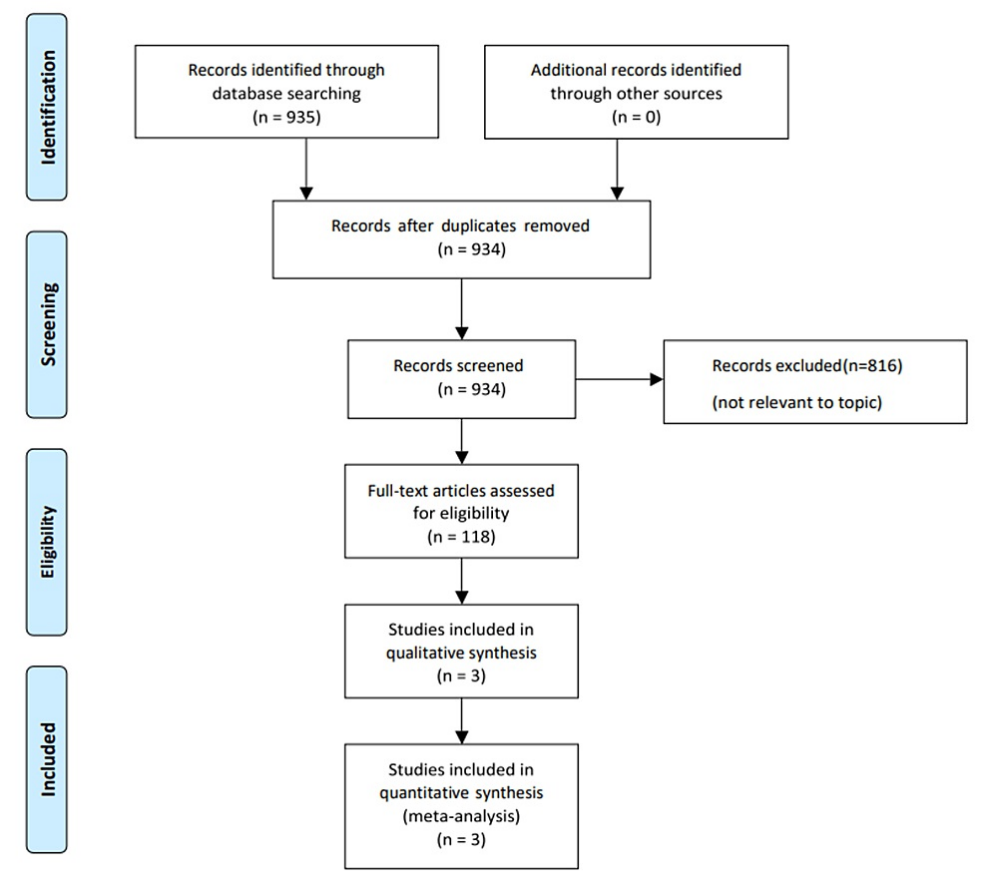
Preferred Reporting Items for Systematic Reviews and Meta-Analyses (PRISMA) flowchart for article screening and selection.

Figure 1 demonstrates the article screening and selection process with three studies meeting the inclusion criteria for quantitative synthesis including 1,400 flaps. Table 1 comprises an amalgamation of the findings from the studies, and Table 2 summarises the findings of the Newcastle-Ottawa scale used to assess the quality of non-randomised studies.

**Table 1:** Demographic information, BMI, smoking, flap type, unilateral or bilateral, neoadjuvant chemotherapy, profile of antibiotics, and regime of the included studies. DIEP = deep inferior epigastric perforator; TRAM = transverse rectus abdominis myocutaneous; SIEA = superficial inferior epigastric perforator; TUG = transverse upper gracilis; IGAP = inferior gluteal artery perforator; SGAP = superior gluteal artery perforator; AB = antibiotic; BMI = body mass index

Study and year	Study design	AB, N			Mean age/years		BMI, n (%)		Smoking, n (%)		Flap type, n (%) ≥24 hrs	Unilateral/Bilateral reconstruction	Neoadjuvant chemotherapy, n (%)		Mean duration of AB prophylaxis (days), mean ± SD		AB regime, %
		≤24 hours		≥24 hours	≤24 hours	≥24 hours	≤24 hours	≥24 hours	≤24 hours	≥24 hours			≤24 hours	≥24 hours	≤24 hours	≤24 hours	
Changchien et al. 2023 [11]	Case series	82	26		44.74 ± 7.72	44.86 ± 12.1	23.06 ± 3.5	23.42 ± 3.96	2 (2.44)	2 (7.69)	DIEP	N/R	16 (19.51)	2 (7.69)	82	26	Cefazoline, 108
Liu et al. 2012 [12]	Cohort study	82	174		48.6 ± 8.2	49.3 ± 8.8	20 (24.3)	50 (28.7)	8 (9.8)	15 (8.6)	DIEP 285 (78.3), TRAM 39 (10.7), SIEA 17 (4.7), TUG 16 (4.4), SGAP 2 (0.5), IGAP 4 (1.1), Other 1(0.3)	152 (59%)/106 (41%)	49 (59.8)	84 (48.3)	1	10.6	Cefazoline, 78; vancomycin, 16; clindamycin, 5; fluoroquinolone, 1
Drury et al. 2016 [13]	Retrospective cohort	659	377		50 ± 9.2	51 ± 9.2	28 ± 5.5	29 ± 5.0	53 (8)	31 (8.2)	Non-latissimus purely autologous, 583 (56.3); latissimus with prosthesis, 453 (43.7)	267	N/R	N/R	N/R	N/R	N/R

**Table 2:** Newcastle-Ottawa scale to assess the quality of non-randomised studies. Note all included studies have a good quality based on this classification.

Study	Selection	Comparability	Exposure
Changchien et al. (2023) [11]	***	**	***
Liu et al. (2012) [12]	***	**	***
Drury et al. (2016) [13]	***	**	***

Description of studies

Changchien et al. [11]

Changchien et al. conducted a cohort study comprising 108 patients undergoing breast reconstruction with a DIEP flap. Patients were divided into three groups based on the duration of antibiotic prophylaxis (one, three, and more than seven days), and the occurrence of SSIs was identified in each group and compared.

Liu et al. [12]

Liu et al. conducted a retrospective review of 256 patients who underwent breast reconstruction over three years following mastectomy. Two groups of patients were compared, with one group receiving ≤24 hours of prophylactic antibiotics and a second group receiving >24 hours of prophylactic antimicrobial therapy.

Drury et al. [13]

Drury et al. conducted a cohort study comprising 1,036 patients who received postoperative prophylactic antibiotics. The patients were divided into two groups receiving either ≤24 hours or >24 hours of antibiotic therapy. Similarly, the incidence of SSI was assessed between the two groups.

There were 1,400 combined cases across the three selected studies. The primary outcome measure was the incidence of SSI in autologous breast reconstruction following mastectomy. In total, 823 (58.8%) patients received ≤24 hours of antibiotics and 577 (41.2%) received >24 hours of prophylactic antibiotics postoperatively. There was a combined SSI incidence of 7.2%. There were 54 SSIs in the ≤24 hours of antibiotics group (6.6%), and 47 in the >24 hours group (8.1%).

**Figure 2:**
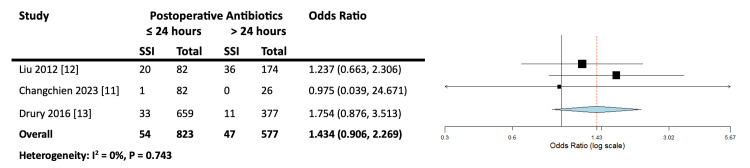
Forest plot for postoperative antibiotic duration on the incidence of SSIs in autologous breast reconstruction following mastectomy. No significant difference between the two groups is observed (OR = 1.434, CI = 0.906, 2.269, p = 0.124). SSI = surgical site infection; OR = odds ratio; CI = confidence interval

There was no significant difference between the two groups (OR = 1.434, CI = 0.906, 2.269, p = 0.124). A low level of heterogeneity was found between the studies (I^2^ = 0%, p = 0.743), as shown in Figure 2.

Discussion

The results of the current review involved the assessment of 1,400 flaps in total both pedicle and free to delineate the effect of postoperative antibiotic duration on the incidence of SSIs post-mastectomy in autologous breast reconstruction. No significant reduction in the incidence of SSIs from prolonged antimicrobial therapy beyond 24 hours was observed. Heterogeneity was negligible on the I^2^ assessment (0%), as well as on the Cochrane Q test (p = 0.74), providing further consistency to the author’s primary outcome measure. A large number of patients in this analysis would suggest that surgeons should avoid antibiotics beyond 24 hours in cases of autologous breast reconstruction to mitigate the caveats of multi-resistant bacterial strains, hospital costs, as well as the adverse outcomes of *Clostridium difficile* infections. Breast surgery is considered to be a clean surgery, and per the Centers for Disease Control and Prevention and the Surgical Care Improvement Project, antibiotic prophylaxis is recommended for a maximum of 24 hours [7]. This has been evidenced by Amland et al. as well as Franchelli et al. who have shown a reduction in the incidence of SSIs in autologous breast reconstruction post-mastectomy when compared to patients who did not receive any antibiotic prophylaxis [14,15]. Studies included within the quantitative assessment of this review demonstrated good comparability for underlying variables. The results of this meta-analysis are comparable to the studies by Phillips et al. and Hai et al. which examined antibiotic prophylaxis in implant-based reconstruction. Both of these studies which included a meta-analysis showed insufficient data to support prolonged antibiotic usage [6,9].

The location of reported SSIs within the studies analysed is generally insufficient. Liu et al. reported a total of 44 (17.1%) SSIs which had an even distribution between the donor site and recipient site, Changchien et al. reported a single SSI (1.2%) which affected the recipient site, and Drury et al. did not mention the site in which SSIs occurred [11-13]. Liu et al. showed insignificant differences in terms of SSI rates for the prolonged antibiotic duration with subgroup analyses including obesity, smoking, and age [12]. Similarly, Drury et al. reported comparable results on subgroup assessments, further enhancing the evidence for abstaining from prolonged antibiotic prophylaxis with sub-cohorts of patients being matched to account for confounding factors. In the same study, Drury et al. examined the difference between the rate of SSIs using different types of flaps including transverse rectus abdominis muscle, latissimus dorsi, and other types of flaps against the duration of antibiotics, which showed no significant difference in prolonged antibiotic usage [13]. Interestingly, the use of fat grafting, another autologous method of breast reconstruction, similarly reports discrepant durations of antibiotic usage in clinical practice with overall low rates of SSIs. This would elicit further clinical assessment to delineate their role including the length of antimicrobial prophylaxis in fat grafting procedures to mitigate the associated drawbacks of antibiotics [16].

The World Health Organization (WHO) does not recommend extending antibiotic duration based on drain placement. This is based on evidence from seven randomised controlled trials which did not show any significant benefits in prolonging antibiotic usage due to the presence of drain in breast and orthopaedic surgery [17]. The main deciding factor in the duration of antibiotics following breast reconstruction has been the placement of drains and the ceasing of antibiotics once drains are removed. The American Association of Plastic Surgeons guidelines state that, in implant-based reconstruction with the absence of drain placement, antibiotics should be stopped within 24 hours. However, if a drain is placed, then it is the surgeon’s preference regarding antibiotic prophylaxis duration [18]. There are risk factors that need to be accounted for to reduce the rate of SSIs in general including tight glycaemic control, normothermia, increased FiO_2_ intraoperative, as well as post-extubation and avoidance of blood transfusion. The up-to-date CDC guidelines suggest an overall incidence rate of SSI to be 1.9% of all surgical procedures in the United States; however, they estimate that this is under-reported [19]. The treatment of SSIs can be costly, both in terms of increased hospital length of stay as well as the treatment required, which is estimated to be between $10,000 and 25,000 [19,20].

Increased antibiotic use comes with complications, both immediate and long-term complications. The use of antibiotics can be associated with adverse events, particularly gastrointestinal symptoms such as nausea, abdominal pain, and diarrhoea. Furthermore, there is an increased risk of developing *Clostridium difficile *infection, which requires treatment with further antibiotics, isolation, and, ultimately, a prolonged hospital stay. Long-term complications mainly constitute the development of antibiotic-resistant organisms making it difficult to treat, as well as reducing the number of available antibiotics in the future [21,22]. The limitations of this review involve the limited number of articles included in the assessment; however, overall the articles included a large number of patients when amalgamated.

This meta-analysis has multiple implications encompassing clinical, financial, and adding to the literature. Limiting prophylactic antibiotic therapy to 24 hours would lead to decreased costs, better human resource management, and decreased antibiotic-associated pathology for autologous breast reconstruction post-mastectomy. The authors conducted a thorough review of the literature with article selection meeting the eligibility criteria of comparing prolonged antibiotic prophylaxis of less than or equal to a 24-hour period. The limitations involve the small number of articles on assessment as well as the observational design of all studies; however, overall, they included a large number of flaps when amalgamated. The conclusion of the review is consistent, with the quantitative assessment of evidence showing no significant reduction in SSI rates from prolonged antimicrobial prophylaxis.

## Conclusions

The results of this meta-analysis indicate that prolonged antibiotic prophylaxis for more than 24 hours in breast reconstruction has an insignificant effect in reducing the incidence of SSIs in autologous breast reconstruction post-mastectomy. Implementing this practice can lead to cost reduction, better allocation of healthcare resources, and reduction in antibiotic-associated complications. Continued research in this field will contribute to refining guidelines and enhancing patient outcomes in autologous breast reconstruction procedures. The authors recommend randomised controlled trials to provide more robust data and account for drain usage in the study design to see if any differences exist in the infection rates.
